# Somatic mutations predict outcomes of hypomethylating therapy in patients with myelodysplastic syndrome

**DOI:** 10.18632/oncotarget.10526

**Published:** 2016-07-11

**Authors:** Seung-Hyun Jung, Yoo-Jin Kim, Seon-Hee Yim, Hye-Jung Kim, Yong-Rim Kwon, Eun-Hye Hur, Bon-Kwan Goo, Yun-Suk Choi, Sug Hyung Lee, Yeun-Jun Chung, Je-Hwan Lee

**Affiliations:** ^1^ Integrated Research Center for Genome Polymorphism, Department of Microbiology, College of Medicine, The Catholic University of Korea, Seoul, Korea; ^2^ Catholic Blood and Marrow Transplantation Center, Seoul St. Mary's Hospital, College of Medicine, The Catholic University of Korea, Seoul, Korea; ^3^ Department of Hematology, Asan Medical Center, University of Ulsan College of Medicine, Seoul, Korea; ^4^ Department of Pathology, College of Medicine, The Catholic University of Korea, Seoul, Korea

**Keywords:** myelodysplastic syndrome, hypomethylating therapy, mutation, targeted sequencing

## Abstract

Although hypomethylating therapy (HMT) is the first line therapy in higher-risk myelodysplastic syndromes (MDS), predicting response to HMT remains an unresolved issue. We aimed to identify mutations associated with response to HMT and survival in MDS. A total of 107 Korean patients with MDS who underwent HMT (57 responders and 50 non-responders) were enrolled. Targeted deep sequencing (median depth of coverage 1,623X) was performed for 26 candidate MDS genes. In multivariate analysis, no mutation was significantly associated with response to HMT, but a lower hemoglobin level (<10g/dL, OR 3.56, 95% CI 1.22-10.33) and low platelet count (<50,000/μL, OR 2.49, 95% CI 1.05-5.93) were independent markers of poor response to HMT. In the subgroup analysis by type of HMT agents, *U2AF1* mutation was significantly associated with non-response to azacitidine, which was consistent in multivariate analysis (OR 14.96, 95% CI 1.67-134.18). Regarding overall survival, mutations in *DNMT1* (*P*=0.031), *DNMT3A* (*P*=0.006), *RAS* (*P*=0.043), and *TP53* (*P*=0.008), and two clinical variables (male-gender, *P*=0.002; IPSS-R H/VH, *P*=0.026) were independent predicting factors of poor prognosis. For AML-free survival, mutations in *DNMT3A* (P<0.001), *RAS* (*P*=0.001), and *TP53* (*P*=0.047), and two clinical variables (male-gender, *P*=0.024; IPSS-R H/VH, *P*=0.005) were independent predicting factors of poor prognosis. By combining these mutations and clinical predictors, we developed a quantitative scoring model for response to azacitidine, overall- and AML-free survival. Response to azacitidine and survival rates became worse significantly with increasing risk-scores. This scoring model can make prognosis prediction more reliable and clinically applicable.

## INTRODUCTION

Myelodysplastic syndromes (MDS) are a group of myeloid neoplasms that are defined by clonal stem cell disorders and characterized by ineffective hematopoiesis and an increased risk of progression to acute myeloid leukemia (AML) [1]. This syndrome shows variable clinical courses, from indolent to life-threatening conditions related to severe cytopenia or progression to AML. Therapy using hypomethylating agents (HMA), such as azacitidine or decitabine, is considered as the first treatment option for patients with lower-risk MDS with significant cytopenia or those with higher-risk MDS [2]. However, it is still unresolved issues for clinicians to predict response to the hypomethylating therapy (HMT) and survival following HMT.

Various prognostic scoring systems have been developed to estimate prognosis which can support decision making for selecting therapeutic options [3]. Among those, the revised International Prognostic Scoring System (IPSS-R), the most recently updated system, is useful to predict survival of patients with or without active treatment including HMT [4–6]. However, the system cannot be used to predict response specifically to HMT [7]. The IPSS-R consists of bone marrow blast percentage, degree of cytopenia, and cytogenetic risk groups. Cytogenetic test results reflect the biological characteristics of MDS cells, but approximately half the patients have cytogenetic abnormalities which makes them less discriminating. Thus, if reliable molecular genetic markers were identified, they may provide additional prognostic information on MDS.

To get a better understanding of MDS pathogenesis, recurrent somatic mutations and their associations with MDS pathophysiology have been under active investigation [8, 9]. With such efforts, some mutations have been added to existing prognostic scoring systems or used to develop new systems based on their independent prognostic implications [10, 11]. With regard to HMT, *TET2*, *DNMT3A* and *ASXL1* mutations have been reported to be associated with treatment response in MDS patients [12–14], but not in chronic myelomonocytic leukemia [15]. In this study, we aimed to discover mutations associated with response to HMT and survival in Korean MDS patients. For this, we analyzed the genomes of MDS patients showing various responses to HMT by targeted deep sequencing.

## RESULTS

### Targeted deep sequencing of MDS genomes

To discover mutations related to response to HMT in MDS patients, 107 MDS patients were analyzed using targeted deep sequencing. For this, MDS patients were categorized into two groups according to their response to HMA (57 responders and 50 non-responders). Their clinicopathological characteristics are shown in Table 1. Targeted deep sequencing was performed using the target gene panel consisting of 26 genes evidently or potentially associated with MDS (*DNMT3A*, *TET2*, *EZH2, RUNX1, ASXL1, STAG2, CBL, TP53, SRSF2, SF3B1, U2AF1, LAMB4, DNMT1, ETV6, KRAS, NF1, NPM1, NRAS, PRPF8, IDH1, IDH2, JAK2, FLT3, SETBP1, ATRX,* and *ZRSR2*) [10–14, 16–20]. The median depth of coverage for the targeted deep sequencing was 1,623x (range 571x-4,437x) across the entire genome (Supplementary Table S1).

**Table 1:** Baseline characteristics and treatment outcomes of study subjects

Clinical characteristics	Total (n=107)	Responder (n=57)	Non-responder (n=50)	*P*
Sex				
Male	67 (62.6%)	32 (56.1%)	35 (70.0%)	0.164
Female	40 (37.4%)	25 (43.9%)	15 (30.0%)	
Age				
<60 years.	59 (55.1%)	27 (47.4%)	32 (64.0%)	0.119
≥60 years.	48 (44.9%)	30 (52.6%)	18 (36.0%)	
WHO classification				
RCUD/RCMD	33 (30.8%)	15 (26.3%)	18 (36.0%)	0.693
RAEB1	23 (21.5%)	14 (24.6%)	9 (18.0%)	
RAEB2	46 (43.0%)	25 (43.9%)	21 (42.0%)	
CMML	5 (4.7%)	3 (5.3%)	2 (4.0%)	
IPSS risk group*				
L/Int-1	43 (40.2%)	22 (38.6%)	21 (42.0%)	0.695
Int-2/H	63 (58.9%)	35 (61.4%)	28 (56.0%)	
IPSS-R risk group*				
VL/L/Int	37 (34.6%)	23 (40.4%)	14 (28.0%)	0.226
H/VH	69 (64.5%)	34 (59.6%)	35 (70.0%)	
Hemoglobin				
<10g/dL	79 (73.8%)	37 (64.9%)	42(84.0%)	0.029
≥10g/dL	28 (26.2%)	20 (35.1%)	8 (16.0%)	
ANC				
<800 cells/μL	39 (36.4%)	20 (35.1%)	19 (38.0%)	0.841
≥800 cells/μL	68 (63.6%)	37 (64.9%)	31 (62.0%)	
Platelets				
<50,000/μL	41 (38.3%)	16 (28.1%)	25 (50.0%)	0.028
≥50,000/μL	66 (61.7%)	41 (71.9%)	25 (50.0%)	
Blasts in BM				
<5%	42 (39.3%)	23 (40.4%)	19 (38.0%)	0.845
≥5%	65 (60.7%)	34 (59.6%)	31 (62.0%)	
Pre-HMA treatment				
None	99 (92.5%)	54 (94.7%)	45 (90.0%)	0.469
EPO/CS/OXM	8 (7.5%)	3 (5.3%)	5 (10.0%)	
HMA				
Azacitidine	66 (61.7%)	40 (70.2%)	26 (52.0%)	0.073
Decitabine	41 (38.3%)	17 (29.8%)	24 (48.0%)	
No of HMA cycles, median (range)	4 (1-18)	6 (1-18)	2 (1-10)	<0.001
Type of best response				
CR	-	12 (21.1%)	-	
mCR±HI	-	28 (49.1%)	-	
SD+HI	-	17 (29.8%)	-	
Type of treatment failure				
DP	-	-	12 (24.0%)	
SD-HI	-	-	33 (66.0%)	
Intolerable/toxic death	-	-	5 (10.0%)	
Overall survival				
No of death	45	19	26	
Probability at 2 years	62.4%	71.8%	51.4%	0.015
AML-free survival				
No. of AML progression	28	12	16	
Probability at 2 years	71.3%	79.2%	61.8%	0.039

* IPSS and IPSS-R data of one patient in non-responder group is not available. N, number; WHO, World Health Organization; RCUD, refractory cytopenia with unilineage dysplasia; RCMD, refractory cytopenia with multilineage dysplasia; RAEB, refractory anemia with excess of blasts; CMML, chronic myelomonocytic leukemia; IPSS, International Prognostic Scoring System; IPSS-R, revised IPSS; ANC, absolute neutrophil count; L, Low; VL, very low; Int, intermediate; H, high; VH, very high; HMA, hypomethylating agent; EPO, erythropoietin; CS, cyclosporine; OXM, oxymetholone; CR, complete remission; mCR, marrow CR; HI, hematological improvement; SD, stable disease

**Figure 1:**
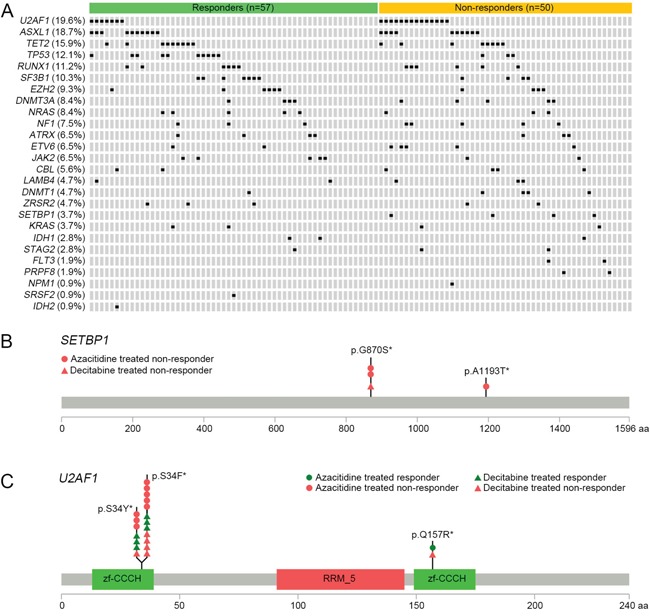
Mutational features of the candidate genes in MDS, and a schematic diagram of *SETBP1* and *U2AF1* mutations **A.** Mutational features of the 26 candidate genes in 107 MDS genomes. Each row represents the mutated gene and each column represents an individual patient. **B.** A diagram of *SETBP1* mutations. Mutation profiles are as follows: G-to-A transitions resulting in p.G870S (n=3) and p. A1193T (n=1). X axis represents amino acid position. Y axis represents the number of mutations. *, Somatic mutations in COSMIC database. **C.** A diagram of *U2AF1* mutations. Mutation profiles are as follows: G-to-A transitions resulting in p.S34F (n=12), G-to-T transversions resulting in p.S34Y (n=7) and T-to-C transitions resulting in p.Q157R (n=2). X axis represents amino acid position. Y axis represents the number of mutations. *, Somatic mutations in COSMIC database.

A majority of the MDS genomes (94/107, 87.9%) had mutations in at least one target gene (Figure 1A, Supplementary Table S2). On average, 1.9 single nucleotide variants (SNVs) and indels were identified per genome (SD 1.4, range 0-7). There were no significant differences in the number and pattern of the mutations between responders (average of 1.7 mutations; 0-5) and non-responders (average of 2.2 mutations; 0-7) (Supplementary Figure S1). Of the mutated genes, six genes were detected in more than 10% of the 107 MDSs; *U2AF1* (19.6%), *ASXL1* (18.7%), *TET2* (15.9%), *TP53* (12.1%), *RUNX1* (11.2%), and *SF3B1* (10.3%). The frequencies of mutations identified in this study were largely similar to those identified in other MDS studies with some exceptions such as *DNMT3A*, *DNMT1, SRSF2, IDH2,* and *NPM1* (Supplementary Table S3) [12, 13, 19–21].

### Factors associated with response to HMT

In univariate analysis, only *SETBP1* mutation was significantly associated with non-response to HMT (mutation frequencies 0% (0/57) in responders vs. 8% (4/50) in non-responders, *P*=0.045) (Table 2, Supplementary Table S4). All *SETBP1* mutations, p.G870S (n=3) and p.A1193T (n=1), identified on exon 4 are missense mutations which are present in the COSMIC database [22] (Figure 1B). Recurrent p.G870S mutation is a hotspot mutation in myeloid neoplasms including MDS [23, 24]. *U2AF1* mutation was also more frequent in non-responders, but the significance was borderline (12.3% in responders vs. 28% in non-responders, *P*=0.052) (Supplementary Table S4). All *U2AF1* mutations, p.S34F (n=12), p.S34Y (n=7) and p.Q157R (n=2), are missense mutations which are present in the COSMIC database (Figure 1C). Among the clinical variables, a lower hemoglobin level (<10 g/dL) and platelet count (<50,000/μL) were significantly associated with non-response (lower hemoglobin, 46.8% in responders vs. 71.4% in non-responders, *P*=0.029; lower platelet, 39.0% vs. 62.1%, *P*=0.028). In multivariate analysis into which the variables with chi-square *P* values < 0.1 were entered, only a hemoglobin level <10g/dL (OR 3.56, 95% CI 1.22-10.33, *P*=0.020) and a platelet count <50,000/μL (OR 2.49, 95% CI 1.05-5.93, *P*=0.039) were found to be associated with positive response to HMT in MDS (Table 2). Although the frequency of *TET2* mutation was similar to those from previous studies conducted in diverse populations (Supplementary Table S3), this mutation was not significantly associated with HMT response in our study. Clinical and genetic variables associated with response to HMT are summarized in Supplementary Table S4.

**Table 2:** Predictive factors of non-response to HMT

Variable	Univariate		Multivariate*	
	*P*	OR (95% CI)	*P*	OR (95% CI)
Clinical variables				
Hemoglobin (< 10g/dL)	0.029	2.84 (1.12-7.20)	0.020	3.56 (1.22-10.33)
Platelets (< 50,000/μL)	0.028	2.56 (1.15-5.71)	0.039	2.49 (1.05-5.93)
Gene mutations				
*U2AF1* mutation	0.052	2.78 (1.02-7.58)	0.138	2.22 (0.77-6.37)
*SETBP1* mutation	0.045	-	0.999	-

* Stepwise multiple logistic regression analysis with the variables of *P*<0.1 in univariate analysis (hemoglobin, platelets, hypomethylating agent and mutations of *TP53, SETBP1,* and *U2AF1* gene) was conducted for multivariable analysis. OR, Odds Ratio of non-response to HMT

Next, we performed the subgroup analysis by HMA type (66 patients treated with azacitidine and 41 treated with decitabine). *U2AF1* mutation was significantly associated with the non-response in the azacitidine group (mutation frequencies 2.5% (1/40) in responders vs. 30.8% (8/26) in non-responders, *P*=0.002) but not in the decitabine group (*P*=0.507). Of note, the eight non-responders in the azacitidine group had p.S34F/Y mutations which are known to be in a mutational hotspot in MDS [25] (Figure 1C). Other frequent mutations in the genes including *TET2*, *ASXL1*, and *RUNX1* did not show any significant associations with response to treatment in both azacitidine and decitabine treated groups. In multivariate analysis conducted in the same manner described above, only *U2AF1* mutation (OR 14.96, 95% CI 1.67-134.18, *P*=0.016) was the independent predictive factor of response to azacitidine in MDS.

### Factors associated with overall and AML-free survival

**Figure 2:**
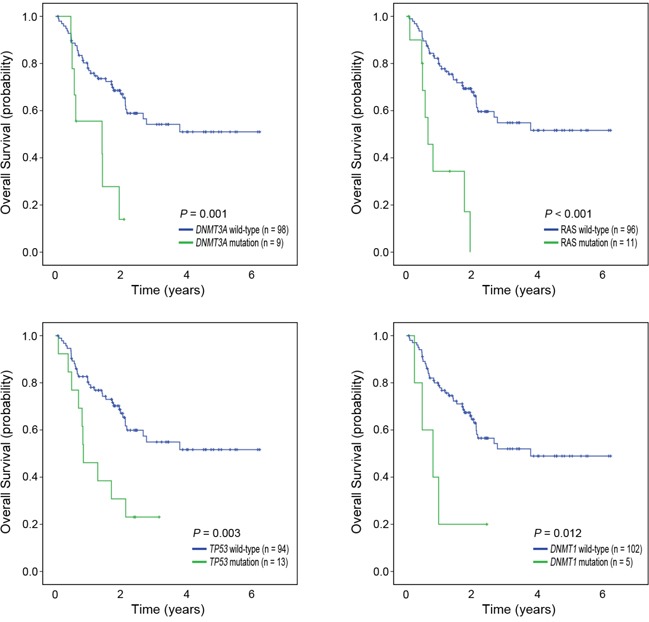
Kaplan-Meier curves for overall survival by mutation status Patients with mutations (green) in *DNMT3A*, *RAS*, *TP53*, and *DNMT1* showed significantly poorer overall survival than the patients without them (blue).

To identify the factors associated with prognosis, survival analysis was performed. The median duration of follow-up was 2.28 years (range 0.07 to 6.24 years) from the start of HMT; 45 patients died and 28 patients progressed to AML. The 2-year overall and AML-free survival rates were 62.4% and 71.3%, respectively. In univariate analysis, six mutations were significantly associated with poorer overall survival (OS) (*DNMT1*, *P*=0.012; *DNMT3A*, *P*=0.001; *TP53*, *P*=0.003; *NPM1*, *P*=0.029; *NRAS*, *P*<0.001; *KRAS*, *P*=0.001) (Supplementary Table S4). When we merged *KRAS* and *NRAS* mutations into *RAS* mutations, OS of the patients with *RAS* mutations were significantly lower than those without them (*P*<0.001) (Supplementary Table S4). In addition to these mutations, five clinical variables (male-gender, *P*=0.006; age ≥60, *P*=0.004; BM blast ≥5%, *P*=0.029; IPSS-R cytogenetic risk poor or very poor, *P*=0.007; IPSS-R high (H) or very high (VH), *P*=0.039) were significantly associated with poorer OS in univariate analysis (Supplementary Table S4). Multivariate analysis with candidate mutations and clinical variables significant in univariate analysis demonstrated that the presence of *DNMT1* (Hazard ratio [HR]=4.08, 95% CI 1.14-14.62, *P*=0.031), *DNMT3A* (HR=4.12, 95% CI 1.51-11.22, *P*=0.006), *RAS* (HR=2.76, 95% CI 1.03-7.37, *P*=0.043), and *TP53* (HR=3.17, 95% CI 1.35-7.43, *P*=0.008) mutations, and two clinical variables (male-gender, HR=3.70, 95% CI 1.63-8.36*, P*=0.002 and IPSS-R H/VH, HR=2.36, 95% CI 1.11-5.02*, P*=0.026) were independent prognostic factors of OS (Table 3). There were no significant differences in the type of HMA and numbers of treatment cycles between those with mutations of four genes and those without them. Significant mutations associated with OS are illustrated in Figure 2.

**Table 3:** Prognostic factors for overall and AML-free survival

	Variable	Univariate*	Multivariate^§^	
		*P*	*P*	HR (95% CI)
Overall survival	Clinical variables			
	Sex (Female vs. Male)	0.006	0.002	3.70 (1.63-8.36)
	IPSS-R (VL/L/Int vs. H/VH)	0.039	0.026	2.36 (1.11-5.02)
	Age (<60 vs. ≥60 years)	0.004	0.073	1.80 (0.95-3.44)
	Gene mutations			
	*DNMT1* (WT vs. MT)	0.012	0.031	4.08 (1.14-14.62)
	*DNMT3A* (WT vs. MT)	0.001	0.006	4.12 (1.51-11.22)
	*RAS* (WT vs. MT)	<0.001	0.043	2.76 (1.03-7.37)
	*TP53* (WT vs. MT)	0.003	0.008	3.17 (1.35-7.43)
AML-free survival	Clinical variables			
	Sex (Female vs. Male)	0.069	0.024	2.85 (1.15-7.09)
	IPSS-R (VL/L/Int vs. H/VH)	0.044	0.005	6.30 (1.77-22.52)
	Gene mutation			
	*DNMT3A* (WT vs. MT)	<0.001	<0.001	12.81 (4.04-40.63)
	*TP53* (WT vs. MT)	0.074	0.047	2.80 (1.01-7.75)
	*RAS* (WT vs. MT)	<0.001	0.001	7.04 (2.24-22.12)

* Univariate survival analysis was performed using the Kaplan-Meier method.

§ Cox proportional hazards model was built with the variables with *P*<0.1 in univariate analysis. IPSS-R, revised International Prognostic Scoring System; VL, very low; L, low; Int, intermediate; H, high; VH, Very High; WT, wild type; MT, mutant type

**Figure 3:**
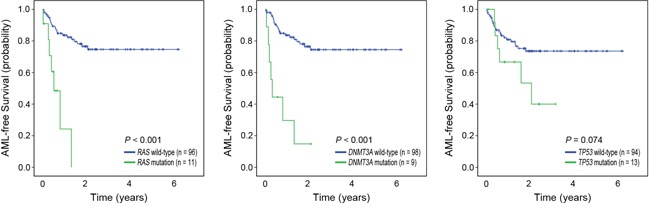
Kaplan-Meier curves for AML-free survival by mutation status Patients with mutations (green) in *DNMT3A, RAS,* and *TP53* showed significantly poorer AML-free survival than the patients without them (blue).

Patients with *DNMT3A* (*P*<0.001), *STAG2* (*P*<0.001), *NPM1* (*P*=0.042) and *NRAS* (*P*<0.001) mutations showed significantly poorer AML-free survival (AFS) in univariate analysis (Supplementary Table S4). *RAS* mutations were also significantly associated with poorer AFS (*P*<0.001). Two clinical variables (BM blast ≥5%, *P*=0.015 and IPSS-R H/VH, *P*=0.044) were significantly associated with poorer AFS in univariate analysis (Supplementary Table S4). Multivariate analysis showed that the presence of *DNMT3A* (HR=12.81, 95% CI 4.04-40.63, *P*<0.001), *TP53* (HR=2.80, 95% CI 1.01-7.75, *P*=0.047), and *RAS* (HR=7.04, 95% CI 2.24-22.12, *P*=0.001) mutations, and two clinical variables (male-gender, HR=2.85, 95% CI 1.15-7.09, *P*=0.024 and IPSS-R H/VH, HR=6.30, 95% CI 1.77-22.52, *P*=0.005) were independent prognostic factors of poorer AFS (Table 3). Significant mutations associated with AFS are illustrated in Figure 3.

### Risk scoring system for predicting treatment response and survival

For the prediction of response to azacitidine, we developed a quantitative scoring model using the clinical and genetic factors that were found to be significantly associated with treatment response (*U2AF1* mutation, hemoglobin level, and platelet count). Taking the ORs from multivariate analysis into consideration, we divided patients into 4 groups; group 1 (hemoglobin ≥10g/dL, platelet count ≥50,000/μL, and *U2AF1* wild-type), group 2 (hemoglobin <10g/dL or platelet count <50,000/μL, and *U2AF1* wild-type), group 3 (hemoglobin <10g/dL, platelet count <50,000/μL, and *U2AF1* wild-type), and group 4 (*U2AF1* mutant-type regardless of clinical factors). The proportions of the HMT responders were significantly different among the groups: 85.7% (12/14) for group 1, 70.0% (21/30) for group 2, 46.2% (6/13) for group 3, and 11.1% (1/9) for group 4 (*P*=0.002).

**Figure 4:**
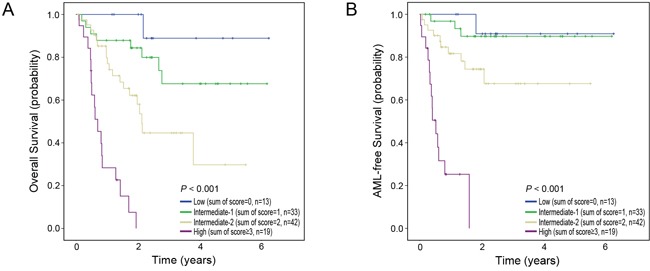
Risk scoring system for predicting survival Kaplan–Meier estimates of overall survival **A.** and AML-free survival **B.** for four risk groups. As sum of scores increased, overall survival and AML-free survival decreased in a score-dependent manner.

We also developed a similar scoring model for predicting survival. Score 1 was assigned respectively to male-gender, IPSS-R H/VH, and each mutation of the four genes (*DNMT1*, *DNMT3A*, *RAS*, and *TP53*) and score 0 to female-gender, IPSS-R VL/L/Int, and wild-type of the four genes. Based on the sum of the scores, we divided patients into four groups; low (score sum=0), intermediate-1 (score sum=1), intermediate-2 (score sum=2) and high (score sum ≥3) risk groups. As the sum of the scores increased, OS (*P*<0.001) and AFS (*P*<0.001) decreased in a score-dependent manner (Figure 4).

## DISCUSSION

Genetic alterations associated with various prognostic factors in MDS have been frequently described [13, 18–20], which implies that mutations in MDS might be used for predicting treatment outcomes. However, the mutation profiles associated with response to HMT have been less well studied [12–15]. The purpose of this study was threefold; first, to reveal mutation profiles of known MDS genes in Korean MDS patients prior to HMT; second, to identify mutations associated with response to HMT and survival; third, to develop a risk scoring system for predicting response to HMT. We adopted two strategies to get more accurate and reliable data. First, to rule out the possibility of contamination with molecular-genetic aberrations acquired following HMT, we only included BM samples collected before starting HMT. Second, we only included the MDS cases that underwent the standard schedule of HMA. The frequencies and overall profiles of mutations identified in this study were largely consistent with previous findings [13, 19–21]. Especially, the mutations of *U2AF1*, *ASXL1* and *TET2* which were detected in over 15% of the patients in this study have also been reported as common in other studies [13, 18–20]. We found one mutation associated with non-response to azacitidine (*U2AF1*) and four mutations associated with poorer OS and/or AFS (*DNMT3A*, *DNMT1*, *TP53*, and *RAS*). By merging significant genetic and clinical factors, we developed risk scoring systems for predicting response to HMT and survival. Considering that *U2AF1* mutation was specifically associated with non-response to azacitidine, our scoring system would be helpful to predict response to azacitidine treatment.

Given the importance of HMA in treating MDS, reliable prediction of patients' response to HMA can be very useful to select treatment options. There have been efforts to identify clinical features which can predict better response to HMT. However, the results have been inconsistent, which hinders clinical application of these features. For example, Itzykson *et al.* investigated 282 high-risk MDS patients who were receiving azacitidine and reported that BM blasts <15%, normal karyotype, and no previous treatments with low-dose cytarabine were associated with better response [7]. Traina *et al.* reported that platelet <100,000/μL predicted poor response [12]. We found that the extent of cytopenia (hemoglobin <10g/dL or platelet <50,000/μL) was associated with poor response to HMT, which is coherent with the observation by Traina *et al.*

Among the genetic predictors of response to HMT, the most commonly mentioned one is *TET2* mutation, though this association has not always been consistent. For example, in several studies, patients with *TET2* mutation showed a higher response rate [12, 14]. In Bejar *et al.*'s study which used the samples collected before HMT, *TET2* mutation predicted positive response to HMT, but only when clonal mutations (VAF >10%) were used [13]. In Braun *et al.*'s study, mutations in leukemia-related genes including *TET2* and *ASXL1* did not predict positive response to HMT in chronic myelomonocytic leukemia (CMML), one of the myelodysplastic/myeloproliferative disorders [15]. In our study, *TET2* mutations did not show any significant associations with treatment response (either to azacitidine or decitabine) even when the confidence mutations (VAF >10%) were used. The discrepancy may be caused by the relatively smaller sample size of this study or population differences. Also, BM sampling time was not consistent in all the studies (for example, in Traina *et al*.'s study, 52% of the samples were collected after HMT and the other 48% were collected prior to HMT [12]), which might also affect the mutation profiles. Further studies with larger samples including matched normal samples are required to verify the implications of the frequent mutations such as *TET2*.

In multivariate analysis, none of the mutations significant in univariate analysis showed associations with response to HMT. However, in the subgroup analysis by type of HMA, *U2AF1* mutation was significantly associated with no-response to azacitidine, which was consistent in multivariate analysis. U2AF1 is a U2 auxiliary factor protein that plays an important role in RNA splicing and *U2AF1* mutations are commonly observed in MDS [13, 25]. To our knowledge, this is the first report of the association between *U2AF1* mutation and response to azacitidine. Of note, all non-responders to azacitidine with *U2AF1* mutation had their mutations in a single hotspot (p.S34F/Y). This mutation induces abnormal splicing of the genes involved in MDS pathogenesis [26, 27]. Ilagan *et al.* found that *U2AF1* mutations caused differential splicing of hundreds of genes including the *DNMT3B* gene [28] which further supports the biological implications of recurrent *U2AF1* mutations on response to azacitidine.

*SETBP1* mutation was significantly associated with no-response to HMT in this study. Although this association was lost in multivariate analysis, somatic mutations in the *SETBP1* gene have been reported to be associated with myeloid malignancies including MDS [23, 24, 29], suggesting the potential implication of the *SETBP1* mutation on HMT response. Especially, *SETBP1* mutations were more prevalent in high-risk MDS (refractory with excess of blasts [RAEB] and secondary AML) and CMML, which suggests their roles in disease progression. Similarly, in this study, *SETBP1* mutations were identified especially in high-risk MDS (two of RAEB-2 and one of CMML).

Regarding the effects of genetic mutations on survival, mutations in *TP53*, *EZH2*, *RUNX1*, *ETV6*, *ASXL1*, *SRSF2*, *U2AF1*, and *SF3B1* have shown prognostic relevance [17, 20, 30–33] and the models composed of the genetic factors and clinical factors outperformed the IPSS-R. In HMT, mutations in *ASXL1*, *TP53*, and *PTPN11* were suggested as markers of inferior survival, whereas *SF3B1* mutation was a marker of favorable survival [12, 13, 34]. In the transplantation setting, mutations in *TP53*, *TET2*, and *DNMT3A* were predictors of poorer survival [19]. Our study showed that mutations in *TP53*, *RAS* (*KRAS* and *NRAS*), *DNMT1*, and *DNMT3A* were independent predictors of poor survival after HMT. Taken our results and previous reports together, *TP53* and *DNMT3* mutations seem to be associated with poorer survival, but the prognostic roles of mutations in other genes, such as *DNMT1, RAS,* and *TET2* remain to be validated.

By merging significant genetic and clinical factors, we developed risk scoring systems for predicting response to azacitidine and survival. Response to azacitidine and survival rates became worse significantly with increasing risk-scores, suggesting that this scoring model can predict the treatment outcomes in more detailed fashion. However, we could not perform an independent validation of our model in this study. Further studies with larger independent samples will be required to validate its clinical validity and applicability.

In summary, this study identified the independent molecular markers for the prediction of response to HMT or survival following it. We discovered a hotspot mutation of the *U2AF1* which was associated with poorer response to azacitidine. Furthermore, the mutations in *TP53*, *RAS*, *DNMT3A*, and *DNMT1* were identified as independent predictors of poorer OS, and DNMT3A, TP53 and RAS mutations as predictors of poorer AFS following HMT. Based on our findings, we developed a quantitative scoring model for response to HMT and survival, which can make prognosis prediction more reliable and clinically applicable.

## MATERIALS AND METHODS

### Study subjects

A total of 107 Korean patients with MDS including CMML were enrolled in this study. The clinical and molecular variables were identified prior to HMT. All patients were treated with one of two HMAs: azacitidine (n=66) or decitabine (n=41). The median age of the cohort was 59 (range 23-76 years). All patients were recruited from the Asan Medical Center (Seoul, Korea) and Seoul St. Mary's Hospital (Seoul, Korea). We only included the patients whose pre-HMT bone marrow (BM) samples were available to avoid potential bias from molecular aberrations acquired during and after HMT. The baseline characteristics and treatment outcomes are shown in Table 1. Responders are more frequent in the azacitidine treated group. Comparisons of baseline characteristics between two groups showed no significant differences but the average age of the patients were higher in the azacitidine group (Supplementary Table S5). Response to treatment was assessed using the modified International Working Group (IWG 2006) response criteria [35]. Patients who achieved complete response (CR), partial response, marrow CR, or stable disease with hematologic improvement (HI) were considered as responders (n=57, 53.3%), and the others as non-responders (n=50, 46.7%). This study was approved by the institutional review board of each institute. Written informed consent was obtained from each patient.

### Targeted deep sequencing

A total of 26 well-known genes in MDS (*DNMT3A, TET2, EZH2, RUNX1, ASXL1, STAG2, CBL, TP53, SRSF2, SF3B1, U2AF1, LAMB4, DNMT1, ETV6, KRAS, NF1, NPM1, NRAS, PRPF8, IDH1, IDH2, JAK2, FLT3, SETBP1, ATRX, and ZRSR2*) were analyzed by targeted deep sequencing in the 107 MDS genomes (57 responders and 50 non-responders). In brief, sequencing libraries were generated using AmpliSeq Library Kit 2.0 with a customized target panel (Life Technologies, Carlsbad, CA) according to the manufacturer's instructions. This customized panel consists of 1,088 amplicons covering 98.4% of all coding exons in 26 target genes. Sequencing was performed on a P1 chip on the Ion Torrent Proton (Life Technologies) according to the manufacturer's instructions. Sequencing reads were aligned to GRCh37/hg19 and genomic variants were called using the Torrent Suite 4.2. The information of sequencing alignments (e.g., the number of reads and sequencing coverage) were summarized in Supplementary Table S1. To discover meaningful mutations, stringent post-filtering processes were conducted. Initially, we selected functional variants in coding exons. Known polymorphic sites (>1% of minor allele frequency) in public databases (dbSNP138, ESP6500, and the 1000 genomes project) were filtered out as polymorphisms. Variants that show >1% of minor allele frequency in our in-house normal database (38 whole genome and 2,283 whole exome sequencing data from Koreans) were also filtered out. Remaining variants were considered candidate somatic mutations.

### Statistical analysis

Categorical variables were compared using the Fisher's exact test or chi-square test as appropriate, and continuous variables were compared using the Student's t test. For survival analysis, time-to-event was defined as duration from the date of HMT to the date of death from any cause OS or the date of AML progression AFS. In univariate survival analysis, survivals were calculated according to the Kaplan-Meier method. Differences in survival curves were assessed with the log-rank test. Stepwise multiple logistic regression and Cox proportional hazards models were used for multivariate analysis. *P* less than 0.05 were considered significant in all statistical analyses.

## SUPPLEMENTARY DATA, FIGURES AND TABLES




