# Supplementary material for: Somatic mutations predict outcomes of hypomethylating therapy in patients with myelodysplastic syndrome
Source: Oncotarget. 2016 Jul 11;7(34):55264–75. doi: 10.18632/oncotarget.10526 (PMC5342416; doi:10.18632/oncotarget.10526)
Supplement: Supplementary file 1 [file oncotarget-07-55264-s001.pdf]

Somatic mutations predict outcomes of hypomethylating therapy in patients with myelodysplastic syndrome

SUPPLEMENTARY DATA

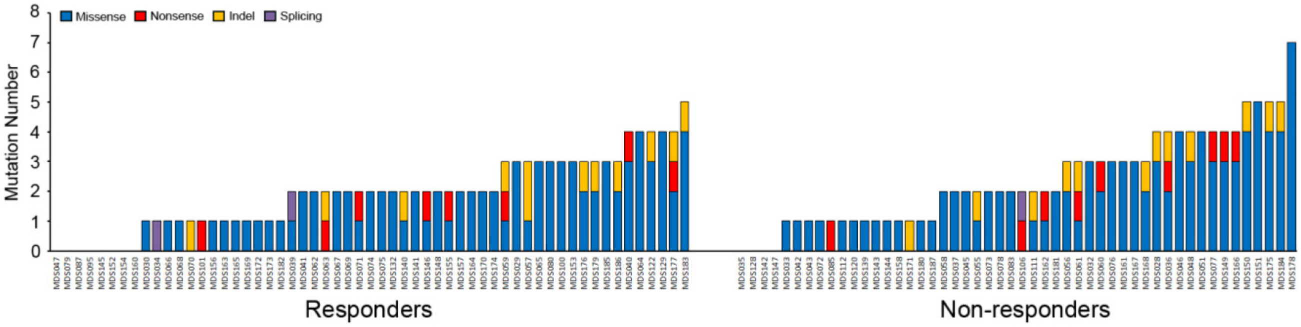

Supplementary Figure S1: Mutational profiles of 107 MDS genomes. The numbers of mutations are shown with the four functional categories indicated in the inset. There were no significant differences in the mutation numbers and mutation profiles between responders (average of 1.7 mutations; 0-5) and non-responders (average of 2.2 mutations; 0-7).

Supplementary Table S1: The description of targeted sequencing data

See Supplementary File 1

Supplementary Table S2: SNVs and indels identified across 107 MDS genomes

See Supplementary File 1

Supplementary Table S3: Comparison of our gene mutations with other MDS cohorts

	Jung et al.§		Bejar et al.[1]	Bejar et al.[2]	Haferlach et al.[3]	Papaemmanuil et al.[4]	Traina et al.[5]
**Method**	Target Sequencing		Target Sequencing	Target Sequencing	Target Sequencing	Target Sequencing	Sanger Sequencing
**Gene number**	26		41	40	104	112	9
**Target Region**	Whole-gene		Hotspot	Hotspot	Whole-gene	Whole-gene	Hotspot
**Sample Size**	107	107 (>10% VAF)	213	87	944	738	92
Mutated Genes							
U2AF1	19.6%	17.8%	13.6%	13.8%	7.7%	6.1%	-
ASXL1	18.7%	15.9%	46.5%	28.7%	23.4%	13.7%	26.1%
TET2	15.9%	11.2%	27.2%	12.6%	33.3%	25.9%	18.5%
TP53	12.1%	11.2%	18.3%	20.7%	6.4%	4.7%	-
RUNX1	11.2%	10.3%	19.7%	16.1%	10.6%	8.1%	-
SF3B1	10.3%	6.5%	15.0%	9.2%	32.9%	24.7%	13.0%
EZH2	9.3%	7.5%	9.9%	3.4%	5.5%	5.3%	-
DNMT3A	8.4%	6.5%	16.0%	18.4%	13.1%	10.6%	8.7%
NRAS	8.4%	6.5%	11.3%	8.0%	3.8%	2.8%	2.2%
NF1	7.5%	1.9%	9.9%	4.6%	3.0%	3.7%	-
ETV6	6.5%	5.6%	3.8%	3.4%	2.3%	1.4%	-
JAK2	6.5%	3.7%	4.7%	5.7%	4.8%	4.5%	-
ATRX	6.5%	0%	5.6%	2.3%	0.8%	2.4%	-
CBL	5.6%	2.8%	6.6%	6.9%	5.1%	4.2%	3.3%
LAMB4	4.7%	3.7%	-	-	2.2%	-	-
DNMT1	4.7%	2.8%	-	-	-	0.9%	-
ZRSR2	4.7%	3.7%	4.7%	5.7%	7.6%	4.5%	-
SETBP1	3.7%	3.7%	-	-	-	-	-
KRAS	3.7%	1.9%	3.8%	3.4%	2.5%	1.6%	-
IDH1	2.8%	0.9%	4.7%	3.4%	2.5%	2.6%	2.2%
STAG2	2.8%	0.9%	-	-	7.5%	4.9%	-
FLT3	1.9%	0.9%	-	1.1%	1.2%	2.0%	-
PRPF8	1.9%	0.9%	4.7%	9.2%	1.2%	-	-
SRSF2	0.9%	0.9%	16.4%	10.3%	17.5%	-	-
IDH2	0.9%	0.9%	7.0%	2.3%	3.9%	4.1%	5.4%
NPM1	0.9%	0.9%	3.8%	-	1.0%	1.2%	-

§ This study.

Supplementary Table S4: Univariate analysis of prognostic factors for clinical outcomes after hypomethylating therapy

See Supplementary File 1

Supplementary Table S5: Comparison of patient characteristics between azacitidine and decitabine groups

Characteristic	Total (n=107)	Azacitidine (n=66)	Decitabine (n=41)	*P*
Sex				
Male	67 (62.6%)	40 (60.6%)	27 (65.9%)	0.682
Female	40 (37.4%)	26 (39.4%)	14 (34.1%)	
Age				
<60 years.	59 (55.1%)	30 (45.5%)	29 (70.7%)	0.016
≥60 years.	48 (44.9%)	36 (54.5%)	12 (29.3%)	
WHO classification				
RCUD/RCMD	33 (30.8%)	22 (33.3%)	11 (26.8%)	0.664
RAEB1	23 (21.5%)	14 (21.2%)	9 (22.0%)	
RAEB2	46 (43.0%)	26 (39.4%)	20 (48.8%)	
CMML	5 (4.7%)	4 (6.1%)	1 (2.4%)	
IPSS risk group				
L/Int-1	43 (40.2%)	28 (42.4%)	15 (37.5%)	0.686
Int-2/H	63 (58.9%)	38 (57.6%)	25 (62.5%)	
IPSS-R risk group				
VL/L/Int	37 (34.6%)	26 (39.4%)	11 (27.5%)	0.293
H/VH	69 (64.5%)	40 (60.6%)	29 (72.5%)	
Hemoglobin				
<10g/dL	79 (73.8%)	45 (68.2%)	34 (82.9%)	0.115
≥10g/dL	28 (26.2%)	21 (31.8%)	7 (17.1%)	
ANC				
<800 cells/μL	39 (36.4%)	22 (33.3%)	17 (41.5%)	0.416
≥800 cells/μL	68 (63.6%)	44 (66.7%)	24 (58.5%)	
Platelets				
<50,000/μL	41 (38.3%)	23 (34.8%)	18 (43.9%)	0.415
≥50,000/μL	66 (61.7%)	43 (65.2%)	23 (56.1%)	
Blasts in BM				
<5%	42 (39.3%)	30 (45.5%)	12 (29.3%)	0.108
≥5%	65 (60.7%)	36 (54.5%)	29 (70.7%)	
Previous treatment				
None	99 (92.5%)	62 (93.9%)	37 (90.2%)	0.708
EPO/CS/OXM	8 (7.5%)	4 (6.1%)	4 (9.8%)	

WHO, World Health Organization; RCUD, refractory cytopenia with unilineage dysplasia; RCMD, refractory cytopenia with multilineage dysplasia; RAEB, refractory anemia with excess of blasts; CMML, chronic myelomonocytic leukemia; IPSS, International Prognostic Scoring System; IPSS-R, revised IPSS; ANC, absolute neutrophil count; L, Low; VL, very low; Int, intermediate; H, high; VH, very high; BM, bone marrow; EPO, erythropoietin; CS, cyclosporine; OXM, oxymetholine

SUPPLEMENTARY REFERENCES

1. Bejar R, Lord A, Stevenson K, et al. TET2 mutations predict response to hypomethylating agents in myelodysplastic syndrome patients. Blood. 2014; 124:2705-2712.
2. Bejar R, Stevenson KE, Caughey B, et al. Somatic mutations predict poor outcome in patients with myelodysplastic syndrome after hematopoietic stem-cell transplantation. J Clin Oncol. 2014; 32:2691-2698.
3. Haferlach T, Nagata Y, Grossmann V, et al. Landscape of genetic lesions in 944 patients with myelodysplastic syndromes. Leukemia. 2014; 28:241-247.
4. Papaemmanuil E, Gerstung M, Malcovati L, et al. Clinical and biological implications of driver mutations in myelodysplastic syndromes. Blood. 2013; 122:3616-3627.
5. Traina F, Visconte V, Elson P, et al. Impact of molecular mutations on treatment response to DNMT inhibitors in myelodysplasia and related neoplasms. Leukemia. 2014; 28:78-87.
