# Supplementary material for: Somatic mutations predict outcomes of hypomethylating therapy in patients with myelodysplastic syndrome
Source: Oncotarget. 2016 Jul 11;7(34):55264–75. doi: 10.18632/oncotarget.10526 (PMC5342416; doi:10.18632/oncotarget.10526)
Supplement: Supplementary file 2 [file oncotarget-07-55264-s002.docx]

**Supplementary Tables**

**Supplementary Table S1. The description of targeted sequencing data**

| Sample ID | Sequencing reads | Mapped  (%) | Coverage  (mean) | % of bases  (>= 20 reads) |
| --- | --- | --- | --- | --- |
| MDS009 | 1,681,224 | 1,665,769 (99.1%) | 1316 | 99.1% |
| MDS019 | 1,800,325 | 1,788,291 (99.3%) | 1312 | 97.7% |
| MDS020 | 1,973,406 | 1,957,809 (99.2%) | 1534 | 99.2% |
| MDS021 | 2,207,013 | 2,187,953 (99.1%) | 1650 | 99.1% |
| MDS028 | 2,043,717 | 2,024,214 (99.0%) | 1532 | 99.4% |
| MDS029 | 1,798,269 | 1,781,571 (99.1%) | 1390 | 99.2% |
| MDS030 | 1,973,096 | 1,956,046 (99.1%) | 1349 | 98.9% |
| MDS032 | 5,508,702 | 5,450,347 (98.9%) | 3580 | 99.2% |
| MDS033 | 1,849,983 | 1,834,138 (99.1%) | 1397 | 99.3% |
| MDS034 | 2,270,833 | 2,248,127 (99.0%) | 1676 | 99.4% |
| MDS035 | 1,830,182 | 1,812,566 (99.0%) | 1661 | 99.2% |
| MDS036 | 3,150,496 | 3,115,081 (98.9%) | 2206 | 99.6% |
| MDS037 | 1,612,341 | 1,597,741 (99.1%) | 2098 | 98.8% |
| MDS039 | 2,059,955 | 2,042,014 (99.1%) | 1884 | 99.1% |
| MDS040 | 1,972,120 | 1,953,754 (99.1%) | 1798 | 99.0% |
| MDS041 | 2,127,961 | 2,109,576 (99.1%) | 1579 | 99.0% |
| MDS042 | 2,185,380 | 2,166,416 (99.1%) | 1552 | 99.1% |
| MDS043 | 1,554,399 | 1,540,274 (99.1%) | 1141 | 98.9% |
| MDS045 | 2,310,246 | 2,286,811 (99.0%) | 1670 | 99.2% |
| MDS046 | 2,346,725 | 2,325,480 (99.1%) | 1743 | 98.9% |
| MDS047 | 1,918,162 | 1,904,268 (99.3%) | 1400 | 98.9% |
| MDS048 | 1,448,257 | 1,435,694 (99.1%) | 1094 | 98.7% |
| MDS051 | 1,409,534 | 1,400,672 (99.4%) | 1079 | 98.8% |
| MDS055 | 2,074,155 | 2,056,876 (99.2%) | 1607 | 99.0% |
| MDS057 | 2,149,508 | 2,132,201 (99.2%) | 1627 | 99.2% |
| MDS060 | 1,722,364 | 1,705,795 (99.0%) | 1298 | 98.8% |
| MDS062 | 1,800,921 | 1,786,957 (99.2%) | 1297 | 98.1% |
| MDS063 | 2,135,490 | 2,117,529 (99.2%) | 1584 | 98.6% |
| MDS064 | 2,768,917 | 2,741,581 (99.0%) | 1887 | 97.8% |
| MDS065 | 1,511,016 | 1,497,740 (99.1%) | 1066 | 97.2% |
| MDS066 | 1,505,136 | 1,492,547 (99.2%) | 1089 | 97.5% |
| MDS067 | 5,311,792 | 5,260,831 (99.0%) | 3701 | 99.0% |
| MDS068 | 1,930,176 | 1,914,450 (99.2%) | 1385 | 98.2% |
| MDS069 | 1,527,385 | 1,516,596 (99.3%) | 1106 | 98.2% |
| MDS070 | 1,707,979 | 1,696,286 (99.3%) | 1225 | 98.2% |
| MDS071 | 3,876,788 | 3,841,221 (99.1%) | 2623 | 98.5% |
| MDS072 | 3,301,249 | 3,272,609 (99.1%) | 2280 | 98.0% |
| MDS073 | 2,399,906 | 2,378,734 (99.1%) | 1667 | 97.7% |
| MDS074 | 2,592,946 | 2,567,067 (99.0%) | 1785 | 98.4% |
| MDS075 | 2,263,760 | 2,247,350 (99.3%) | 1623 | 98.6% |
| MDS076 | 4,029,987 | 3,998,129 (99.2%) | 2715 | 98.5% |
| MDS077 | 3,918,313 | 3,882,584 (99.1%) | 2669 | 98.6% |
| MDS078 | 1,400,289 | 1,386,926 (99.0%) | 1046 | 98.4% |
| MDS079 | 1,555,884 | 1,543,642 (99.2%) | 1139 | 98.0% |
| MDS080 | 1,586,986 | 1,575,957 (99.3%) | 1162 | 98.3% |
| MDS083 | 1,440,915 | 1,428,647 (99.1%) | 1049 | 98.1% |
| MDS085 | 1,826,778 | 1,811,931 (99.2%) | 1424 | 98.2% |
| MDS087 | 3,516,007 | 3,483,657 (99.1%) | 2798 | 99.6% |
| MDS095 | 3,590,171 | 3,557,847 (99.1%) | 2921 | 99.6% |
| MDS100 | 2,195,018 | 2,174,292 (99.1%) | 1741 | 99.2% |
| MDS101 | 2,909,998 | 2,886,218 (99.2%) | 2375 | 99.6% |
| MDS106 | 3,239,348 | 3,203,758 (98.9%) | 2541 | 99.4% |
| MDS111 | 3,847,152 | 3,815,666 (99.2%) | 2993 | 99.5% |
| MDS112 | 4,873,383 | 4,833,978 (99.2%) | 3894 | 99.5% |
| MDS120 | 2,379,177 | 2,357,130 (99.1%) | 1920 | 99.0% |
| MDS122 | 2,125,121 | 2,103,673 (99.0%) | 1650 | 98.5% |
| MDS128 | 1,882,552 | 1,867,623 (99.2%) | 1415 | 98.4% |
| MDS129 | 2,059,046 | 2,041,294 (99.1%) | 1556 | 98.6% |
| MDS132 | 2,595,294 | 2,569,820 (99.0%) | 1941 | 99.0% |
| MDS139 | 1,971,875 | 1,952,936 (99.0%) | 1536 | 99.0% |
| MDS140 | 2,001,574 | 1,986,087 (99.2%) | 1454 | 98.6% |
| MDS141 | 1,574,386 | 1,558,586 (99.0%) | 1214 | 98.7% |
| MDS142 | 1,513,768 | 1,496,831 (98.9%) | 1165 | 98.6% |
| MDS143 | 2,203,212 | 2,182,382 (99.1%) | 1357 | 98.7% |
| MDS144 | 2,930,143 | 2,896,444 (98.8%) | 2447 | 99.2% |
| MDS145 | 2,811,872 | 2,787,704 (99.1%) | 1199 | 99.0% |
| MDS146 | 2,506,400 | 2,485,622 (99.2%) | 1425 | 98.9% |
| MDS147 | 2,447,614 | 2,421,285 (98.9%) | 1378 | 99.0% |
| MDS148 | 1,629,310 | 1,614,986 (99.1%) | 1227 | 98.2% |
| MDS149 | 4,201,274 | 4,149,554 (98.8%) | 3040 | 99.0% |
| MDS150 | 4,603,749 | 4,549,881 (98.8%) | 3291 | 98.7% |
| MDS151 | 1,322,806 | 1,311,629 (99.2%) | 1003 | 97.8% |
| MDS152 | 3,082,697 | 3,048,018 (98.9%) | 2164 | 98.6% |
| MDS153 | 6,190,690 | 6,117,593 (98.8%) | 4437 | 99.2% |
| MDS154 | 3,074,934 | 3,046,801 (99.1%) | 2267 | 98.8% |
| MDS155 | 5,172,818 | 5,123,813 (99.1%) | 3725 | 99.1% |
| MDS156 | 1,743,462 | 1,731,694 (99.3%) | 1323 | 98.3% |
| MDS157 | 5,232,514 | 5,164,617 (98.7%) | 3695 | 99.0% |
| MDS158 | 1,322,511 | 1,309,659 (99.0%) | 983 | 98.0% |
| MDS160 | 1,694,626 | 1,682,681 (99.3%) | 1348 | 98.6% |
| MDS161 | 1,387,405 | 1,377,059 (99.3%) | 1102 | 98.7% |
| MDS162 | 2,582,209 | 2,556,412 (99.0%) | 1982 | 99.4% |
| MDS163 | 2,416,375 | 2,394,196 (99.1%) | 1885 | 99.1% |
| MDS164 | 2,041,538 | 2,024,379 (99.2%) | 1556 | 99.0% |
| MDS165 | 4,065,662 | 4,031,099 (99.1%) | 3153 | 99.5% |
| MDS166 | 2,661,593 | 2,636,250 (99.0%) | 1994 | 99.4% |
| MDS167 | 4,000,604 | 3,959,815 (99.0%) | 3008 | 99.5% |
| MDS168 | 2,037,371 | 2,016,774 (99.0%) | 1554 | 99.2% |
| MDS169 | 3,370,227 | 3,330,427 (98.8%) | 2510 | 99.6% |
| MDS170 | 2,146,410 | 2,127,339 (99.1%) | 1668 | 99.3% |
| MDS171 | 3,681,186 | 3,654,130 (99.3%) | 2893 | 99.4% |
| MDS172 | 1,778,151 | 1,765,996 (99.3%) | 1409 | 98.8% |
| MDS173 | 2,567,487 | 2,548,597 (99.3%) | 2047 | 99.2% |
| MDS174 | 2,526,317 | 2,505,588 (99.2%) | 1984 | 99.3% |
| MDS175 | 3,069,104 | 3,043,913 (99.2%) | 2403 | 99.2% |
| MDS176 | 1,141,363 | 1,132,664 (99.2%) | 571 | 98.5% |
| MDS177 | 2,311,435 | 2,293,449 (99.2%) | 1832 | 99.1% |
| MDS178 | 2,469,136 | 2,447,589 (99.1%) | 1888 | 99.3% |
| MDS179 | 1,675,266 | 1,662,903 (99.3%) | 1304 | 98.9% |
| MDS180 | 2,077,649 | 2,060,170 (99.2%) | 1613 | 98.9% |
| MDS181 | 2,167,792 | 2,150,802 (99.2%) | 1698 | 99.2% |
| MDS182 | 2,091,899 | 2,075,515 (99.2%) | 1656 | 99.2% |
| MDS183 | 2,273,954 | 2,256,620 (99.2%) | 1806 | 99.1% |
| MDS184 | 2,004,525 | 1,989,386 (99.2%) | 1581 | 99.0% |
| MDS185 | 1,488,612 | 1,476,216 (99.2%) | 1163 | 98.5% |
| MDS186 | 1,925,745 | 1,909,365 (99.1%) | 1503 | 98.6% |
| MDS187 | 1,742,917 | 1,728,179 (99.2%) | 1356 | 97.4% |

**Supplementary Table S2. SNVs and indels identified across 107 MDS genomes**

| Sample_ID | Position | REF | ALT | Gene | AA_Change* | Type |
| --- | --- | --- | --- | --- | --- | --- |
| MDS077 | chr4:106155928 | G | A | TET2 | p.A277T | Missense |
| MDS167 | chr4:106155928 | G | A | TET2 | p.A277T | Missense |
| MDS061 | chr4:106156201 | G | T | TET2 | p.E368X | Nonsense |
| MDS148 | chr4:106157539 | C | T | TET2 | p.R814C | Missense |
| MDS041 | chr4:106157600 | G | A | TET2 | p.C834Y | Missense |
| MDS149 | chr4:106164787 | C | T | TET2 | p.H1219Y | Missense |
| MDS045 | chr4:106180862 | G | A | TET2 | p.G1297E | Missense |
| MDS174 | chr4:106180865 | G | A | TET2 | p.C1298Y | Missense |
| MDS177 | chr4:106190781 | CAG | C | TET2 | p.H1353fs | frameshift |
| MDS059 | chr4:106193892 | C | T | TET2 | p.R1452X | Nonsense |
| MDS149 | chr4:106194010 | A | G | TET2 | p.K1491R | Missense |
| MDS176 | chr4:106196232 | T | TC | TET2 | p.M1522fs | frameshift |
| MDS185 | chr4:106196238 | A | T | TET2 | p.Q1524L | Missense |
| MDS122 | chr4:106196313 | C | A | TET2 | p.P1549Q | Missense |
| MDS151 | chr4:106196529 | T | C | TET2 | p.L1621P | Missense |
| MDS129 | chr4:106196529 | T | C | TET2 | p.L1621P | Missense |
| MDS149 | chr4:106196657 | C | T | TET2 | p.Q1664X | Nonsense |
| MDS106 | chr4:106197207 | G | A | TET2 | p.W1847X | Nonsense |
| MDS046 | chr4:106197401 | C | G | TET2 | p.H1912D | Missense |
| MDS185 | chr4:106197654 | G | T | TET2 | p.G1996V | Missense |
| MDS078 | chr20:31020730 | A | G | ASXL1 | p.K343E | Missense |
| MDS061 | chr20:31020734 | A | G | ASXL1 | p.E344G | Missense |
| MDS078 | chr20:31020755 | A | G | ASXL1 | p.K351R | Missense |
| MDS071 | chr20:31021211 | C | T | ASXL1 | p.R404X | Nonsense |
| MDS039 | chr20:31022234 | G | A | ASXL1 |  | Splicing |
| MDS150 | chr20:31022364 | A | AT | ASXL1 | p.K618fs | frameshift |
| MDS059 | chr20:31022402 | TCACCACTGCCATAGAGAGGCGGC | T | ASXL1 | p.H630fs | frameshift |
| MDS162 | chr20:31022415 | A | T | ASXL1 | p.R634X | Nonsense |
| MDS167 | chr20:31022445 | G | A | ASXL1 | p.G644R | Missense |
| MDS155 | chr20:31022581 | C | G | ASXL1 | p.S689X | Nonsense |
| MDS146 | chr20:31022592 | C | T | ASXL1 | p.R693X | Nonsense |
| MDS056 | chr20:31022837 | AT | A | ASXL1 | p.L775fs | frameshift |
| MDS036 | chr20:31022837 | AT | A | ASXL1 | p.L775fs | frameshift |
| MDS040 | chr20:31022853 | C | T | ASXL1 | p.Q780X | Nonsense |
| MDS175 | chr20:31022872 | GA | G | ASXL1 | p.T787fs | frameshift |
| MDS186 | chr20:31022936 | TC | T | ASXL1 | p.P808fs | frameshift |
| MDS179 | chr20:31023076 | CTGAT | C | ASXL1 | p.D855fs | frameshift |
| MDS063 | chr20:31023408 | C | T | ASXL1 | p.R965X | Nonsense |
| MDS166 | chr20:31024770 | A | T | ASXL1 | p.K1419X | Nonsense |
| MDS060 | chr20:31024794 | C | T | ASXL1 | p.P1427S | Missense |
| MDS163 | chr20:31024794 | C | T | ASXL1 | p.P1427S | Missense |
| MDS056 | chr7:107696450 | A | G | LAMB4 | p.C1128R | Missense |
| MDS169 | chr7:107698340 | C | T | LAMB4 | p.G1106S | Missense |
| MDS178 | chr7:107710279 | C | A | LAMB4 | p.D726Y | Missense |
| MDS040 | chr7:107718751 | T | A | LAMB4 | p.D633V | Missense |
| MDS083 | chr7:107748257 | C | T | LAMB4 | p.R137Q | Missense |
| MDS166 | chr7:148506413 | C | T | EZH2 | p.C700Y | Missense |
| MDS165 | chr7:148506413 | C | T | EZH2 | p.C700Y | Missense |
| MDS076 | chr7:148506443 | C | T | EZH2 | p.R690H | Missense |
| MDS062 | chr7:148507466 | T | C | EZH2 | p.Y663C | Missense |
| MDS080 | chr7:148508788 | C | T | EZH2 | p.V626M | Missense |
| MDS065 | chr7:148511154 | C | T | EZH2 | R583Q | Missense |
| MDS132 | chr7:148511190 | C | A | EZH2 | p.C571G | Missense |
| MDS073 | chr7:148512065 | G | A | EZH2 | p.S538L | Missense |
| MDS028 | chr7:148523578 | T | C | EZH2 | p.Y292C | Missense |
| MDS101 | chr7:148523591 | G | A | EZH2 | p.R288X | Nonsense |
| MDS076 | chr7:148523657 | C | T | EZH2 | p.G266R | Missense |
| MDS045 | chr11:119142569 | T | G | CBL | p.W190G | Missense |
| MDS064 | chr11:119148881 | A | T | CBL | p.Q367H | Missense |
| MDS129 | chr11:119148931 | G | A | CBL | p.C384Y | Missense |
| MDS064 | chr11:119148982 | G | A | CBL | p.C401Y | Missense |
| MDS150 | chr11:119149241 | C | T | CBL | p.P417S | Missense |
| MDS150 | chr11:119149242 | C | T | CBL | p.P417L | Missense |
| MDS046 | chr11:119149251 | G | A | CBL | p.R420Q | Missense |
| MDS032 | chr11:119155728 | C | T | CBL | p.P494L | Missense |
| MDS161 | chr2:198266834 | T | C | SF3B1 | p.K700E | Missense |
| MDS166 | chr2:198266834 | T | C | SF3B1 | p.K700E | Missense |
| MDS178 | chr2:198266834 | T | C | SF3B1 | p.K700E | Missense |
| MDS029 | chr2:198266834 | T | C | SF3B1 | p.K700E | Missense |
| MDS074 | chr2:198266834 | T | C | SF3B1 | p.K700E | Missense |
| MDS080 | chr2:198266834 | T | C | SF3B1 | p.K700E | Missense |
| MDS157 | chr2:198266841 | C | A | SF3B1 | p.E697D | Missense |
| MDS153 | chr2:198267352 | G | C | SF3B1 | p.Q669E | Missense |
| MDS037 | chr2:198267360 | T | A | SF3B1 | p.K666M | Missense |
| MDS156 | chr2:198267360 | T | G | SF3B1 | p.K666T | Missense |
| MDS100 | chr2:198269858 | T | C | SF3B1 | p.E494G | Missense |
| MDS058 | chr18:42531913 | G | A | SETBP1 | p.G870S | Missense |
| MDS046 | chr18:42531913 | G | A | SETBP1 | p.G870S | Missense |
| MDS175 | chr18:42531913 | G | A | SETBP1 | p.G870S | Missense |
| MDS187 | chr18:42532882 | G | A | SETBP1 | p.A1193T | Missense |
| MDS178 | chr19:10254520 | T | C | DNMT1 | p.K997R | Missense |
| MDS151 | chr19:10257031 | A | G | DNMT1 | p.F948L | Missense |
| MDS037 | chr19:10257188 | G | C | DNMT1 | p.S895R | Missense |
| MDS139 | chr19:10264989 | G | A | DNMT1 | p.R651W | Missense |
| MDS157 | chr19:10273344 | T | C | DNMT1 | p.K320R | Missense |
| MDS184 | chr21:36164585 | CGGCAGGAT | C | RUNX1 | p.I428fs | frameshift |
| MDS184 | chr21:36164596 | G | C | RUNX1 | p.R427G | Missense |
| MDS184 | chr21:36164598 | G | T | RUNX1 | p.P426Q | Missense |
| MDS080 | chr21:36164601 | G | A | RUNX1 | p.P425L | Missense |
| MDS155 | chr21:36164601 | G | A | RUNX1 | p.P425L | Missense |
| MDS140 | chr21:36164706 | T | TG | RUNX1 | p.Q390fs | frameshift |
| MDS183 | chr21:36164722 | AG | A | RUNX1 | p.Y385fs | frameshift |
| MDS055 | chr21:36171612 | G | GA | RUNX1 | p.S318fs | frameshift |
| MDS057 | chr21:36206710 | GC | G | RUNX1 | p.M267fs | frameshift |
| MDS057 | chr21:36206715 | T | C | RUNX1 | p.Q266R | Missense |
| MDS036 | chr21:36231774 | G | A | RUNX1 | p.R204X | Nonsense |
| MDS059 | chr21:36252882 | G | T | RUNX1 | p.D160E | Missense |
| MDS083 | chr21:36252940 | G | A | RUNX1 | p.S141L | Missense |
| MDS151 | chr21:36259172 | G | A | RUNX1 | p.R107C | Missense |
| MDS048 | chr21:36259179 | CG | C | RUNX1 | p.T104fs | frameshift |
| MDS063 | chrX:15809110 | AG | A | ZRSR2 | p.Q32fs | frameshift |
| MDS074 | chrX:15821840 | G | A | ZRSR2 | p.R78K | Missense |
| MDS060 | chrX:15822297 | C | T | ZRSR2 | p.R126X | Nonsense |
| MDS174 | chrX:15827438 | A | G | ZRSR2 | p.D185G | Missense |
| MDS028 | chrX:15833959 | CTA | C | ZRSR2 | p.F239fs | frameshift |
| MDS028 | chrX:15833993 | G | A | ZRSR2 | p.G251R | Missense |
| MDS178 | chrX:76907734 | C | T | ATRX | p.G1476D | Missense |
| MDS066 | chrX:76912053 | G | A | ATRX | p.T1404I | Missense |
| MDS111 | chrX:76919032 | A | AT | ATRX | p.V1320fs | frameshift |
| MDS171 | chrX:76919032 | A | AT | ATRX | p.V1320fs | frameshift |
| MDS122 | chrX:76919032 | A | AT | ATRX | p.V1320fs | frameshift |
| MDS122 | chrX:76937798 | G | T | ATRX | p.Q984K | Missense |
| MDS178 | chrX:76938850 | T | G | ATRX | p.N633T | Missense |
| MDS069 | chrX:76939207 | T | C | ATRX | p.D514G | Missense |
| MDS153 | chrX:76939626 | T | G | ATRX | p.L374F | Missense |
| MDS058 | chr2:25457242 | C | T | DNMT3A | p.R882H | Missense |
| MDS036 | chr2:25457242 | C | T | DNMT3A | p.R882H | Missense |
| MDS051 | chr2:25457242 | C | T | DNMT3A | p.R882H | Missense |
| MDS183 | chr2:25457242 | C | T | DNMT3A | p.R882H | Missense |
| MDS106 | chr2:25457291 | T | C | DNMT3A |  | Splicing |
| MDS170 | chr2:25463247 | C | T | DNMT3A | p.R749H | Missense |
| MDS164 | chr2:25463289 | T | C | DNMT3A | p.Y735C | Missense |
| MDS075 | chr2:25469946 | G | A | DNMT3A | p.R366C | Missense |
| MDS077 | chr2:25472564 | C | T | DNMT3A | p.G12R | Missense |
| MDS051 | chrX:123171446 | T | C | STAG2 | p.F120L | Missense |
| MDS075 | chrX:123195171 | A | G | STAG2 | p.E505G | Missense |
| MDS168 | chrX:123202421 | T | TG | STAG2 | p.L758fs | frameshift |
| MDS179 | chr17:7574033 | T | G | TP53 | p.I332L | Missense |
| MDS029 | chr17:7577096 | T | A | TP53 | p.D281V | Missense |
| MDS112 | chr17:7577100 | T | C | TP53 | p.R280G | Missense |
| MDS129 | chr17:7577105 | G | A | TP53 | p.P278L | Missense |
| MDS172 | chr17:7577539 | G | A | TP53 | p.R248W | Missense |
| MDS151 | chr17:7578203 | C | T | TP53 | p.V216M | Missense |
| MDS161 | chr17:7578283 | G | A | TP53 | p.A189V | Missense |
| MDS161 | chr17:7578394 | T | C | TP53 | p.H179R | Missense |
| MDS186 | chr17:7578395 | G | A | TP53 | p.H179Y | Missense |
| MDS034 | chr17:7578556 | T | C | TP53 |  | Splicing |
| MDS039 | chr17:7579705 | C | T | TP53 | p.V31I | Missense |
| MDS176 | chr17:7579705 | C | T | TP53 | p.V31I | Missense |
| MDS100 | chr17:7579882 | C | G | TP53 | p.E11Q | Missense |
| MDS182 | chr17:7579882 | C | G | TP53 | p.E11Q | Missense |
| MDS178 | chr17:29541524 | A | G | NF1 | p.D483G | Missense |
| MDS122 | chr17:29541524 | A | G | NF1 | p.D483G | Missense |
| MDS048 | chr17:29562973 | C | G | NF1 | p.P1303R | Missense |
| MDS166 | chr17:29663764 | A | C | NF1 | p.M2066L | Missense |
| MDS178 | chr17:29663764 | A | C | NF1 | p.M2066L | Missense |
| MDS180 | chr17:29663764 | A | C | NF1 | p.M2066L | Missense |
| MDS184 | chr17:29663764 | A | C | NF1 | p.M2066L | Missense |
| MDS183 | chr17:29663764 | A | C | NF1 | p.M2066L | Missense |
| MDS070 | chr17:29684303 | A | AT | NF1 | p.T2609fs | frameshift |
| MDS150 | chr21:44514777 | T | C | U2AF1 | p.Q157R | Missense |
| MDS146 | chr21:44514777 | T | C | U2AF1 | p.Q157R | Missense |
| MDS033 | chr21:44524456 | G | T | U2AF1 | p.S34Y | Missense |
| MDS048 | chr21:44524456 | G | T | U2AF1 | p.S34Y | Missense |
| MDS077 | chr21:44524456 | G | T | U2AF1 | p.S34Y | Missense |
| MDS167 | chr21:44524456 | G | T | U2AF1 | p.S34Y | Missense |
| MDS041 | chr21:44524456 | G | T | U2AF1 | p.S34Y | Missense |
| MDS064 | chr21:44524456 | G | T | U2AF1 | p.S34Y | Missense |
| MDS141 | chr21:44524456 | G | T | U2AF1 | p.S34Y | Missense |
| MDS056 | chr21:44524456 | G | A | U2AF1 | p.S34F | Missense |
| MDS055 | chr21:44524456 | G | A | U2AF1 | p.S34F | Missense |
| MDS120 | chr21:44524456 | G | A | U2AF1 | p.S34F | Missense |
| MDS144 | chr21:44524456 | G | A | U2AF1 | p.S34F | Missense |
| MDS158 | chr21:44524456 | G | A | U2AF1 | p.S34F | Missense |
| MDS168 | chr21:44524456 | G | A | U2AF1 | p.S34F | Missense |
| MDS175 | chr21:44524456 | G | A | U2AF1 | p.S34F | Missense |
| MDS181 | chr21:44524456 | G | A | U2AF1 | p.S34F | Missense |
| MDS184 | chr21:44524456 | G | A | U2AF1 | p.S34F | Missense |
| MDS040 | chr21:44524456 | G | A | U2AF1 | p.S34F | Missense |
| MDS065 | chr21:44524456 | G | A | U2AF1 | p.S34F | Missense |
| MDS179 | chr21:44524456 | G | A | U2AF1 | p.S34F | Missense |
| MDS132 | chr12:12022466 | G | T | ETV6 | p.R191L | Missense |
| MDS077 | chr12:12022585 | G | T | ETV6 | p.E231X | Nonsense |
| MDS175 | chr12:12037391 | T | A | ETV6 | p.L341H | Missense |
| MDS085 | chr12:12037492 | G | T | ETV6 | p.G375X | Nonsense |
| MDS177 | chr12:12037501 | C | T | ETV6 | p.R378X | Nonsense |
| MDS048 | chr12:12037507 | T | G | ETV6 | p.W380G | Missense |
| MDS036 | chr12:12038897 | C | T | ETV6 | p.A397V | Missense |
| MDS043 | chr9:5073770 | G | T | JAK2 | p.V617F | Missense |
| MDS060 | chr9:5073770 | G | T | JAK2 | p.V617F | Missense |
| MDS069 | chr9:5073770 | G | T | JAK2 | p.V617F | Missense |
| MDS148 | chr9:5073770 | G | T | JAK2 | p.V617F | Missense |
| MDS029 | chr9:5077540 | T | C | JAK2 | p.L651P | Missense |
| MDS067 | chr9:5080672 | T | G | JAK2 | p.L808W | Missense |
| MDS068 | chr9:5123047 | T | A | JAK2 | p.S1035T | Missense |
| MDS051 | chr13:28592642 | C | A | FLT3 | p.D835Y | Missense |
| MDS072 | chr13:28602329 | G | A | FLT3 | p.A680V | Missense |
| MDS143 | chr17:1563731 | A | G | PRPF8 | p.C1594R | Missense |
| MDS111 | chr17:1576400 | G | A | PRPF8 | p.A1250V | Missense |
| MDS061 | chr5:170832330 | G | GA | NPM1 | p.T234fs | frameshift |
| MDS057 | chr17:74732959 | G | GGGC | SRSF2 | p.P95fs | nonframeshift |
| MDS064 | chr15:90633724 | C | G | IDH2 | p.E120D | Missense |
| MDS067 | chr2:209113112 | C | T | IDH1 | p.R132H | Missense |
| MDS032 | chr2:209113113 | G | A | IDH1 | p.R132C | Missense |
| MDS170 | chr2:209113113 | G | A | IDH1 | p.R132C | Missense |
| MDS183 | chr12:25380276 | T | C | KRAS | p.Q61R | Missense |
| MDS042 | chr12:25398281 | C | T | KRAS | p.G13D | Missense |
| MDS168 | chr12:25398284 | C | A | KRAS | p.G12V | Missense |
| MDS177 | chr12:25398284 | C | A | KRAS | p.G12V | Missense |
| MDS151 | chr1:115256530 | G | T | NRAS | p.Q61K | Missense |
| MDS173 | chr1:115256538 | G | A | NRAS | p.T58I | Missense |
| MDS183 | chr1:115258744 | C | A | NRAS | p.G13V | Missense |
| MDS150 | chr1:115258744 | C | T | NRAS | p.G13D | Missense |
| MDS051 | chr1:115258747 | C | T | NRAS | p.G12D | Missense |
| MDS076 | chr1:115258747 | C | T | NRAS | p.G12D | Missense |
| MDS129 | chr1:115258747 | C | T | NRAS | p.G12D | Missense |
| MDS177 | chr1:115258747 | C | T | NRAS | p.G12D | Missense |
| MDS164 | chr1:115258748 | C | A | NRAS | p.G12C | Missense |

* Amino acid change

**Supplementary Table S4. Univariate analysis of prognostic factors for clinical outcomes after hypomethylating therapy**

| Variable (No. patients) | Response to HMT | *P* | Overall survival  (2-year) | *P* | AML free survival  (2-year) | *P* |
| --- | --- | --- | --- | --- | --- | --- |
| Total patients (107) | 53.3% |  | 62.4% |  | 71.3% |  |
| Clinical variables |  |  |  |  |  |  |
| Sex, male *v* female (67 *v* 40) | 47.8% *v* 62.5% | 0.164 | 52.3% *v* 78.6% | **0.006** | 64.9% *v* 82.0% | **0.069** |
| Age, <60 *v* ≥60 years (59 *v* 48) | 45.8% *v* 62.5% | 0.119 | 73.6% *v* 48.2% | **0.004** | 78.5% *v* 60.6% | 0.123 |
| ANC <800 *v* ≥800/μL (39 *v* 68) | 51.3% *v* 54.4% | 0.841 | 64.5% *v* 61.5% | 0.735 | 74.4% *v* 69.9% | 0.819 |
| Hb <10 *v* ≥10 g/dL (79 *v* 28) | 46.8% *v* 71.4% | **0.029** | 61.2% *v* 65.6% | 0.609 | 70.7% *v* 73.0% | 0.806 |
| PLT <50 *v* ≥50/μL (41 *v* 66) | 39.0% *v* 62.1% | **0.028** | 50.0% *v* 70.3% | 0.215 | 66.6% *v* 73.4% | 0.746 |
| BM blasts <5 *v* ≥5% (42 *v* 65) | 54.8% *v* 52.3% | 0.845 | 75.0% *v* 52.8% | **0.029** | 83.2% *v* 63.3% | **0.015** |
| IPSS-R cytogenetic risk,  VG/G/INT *v* P/VP (82 *v* 25) | 53.7% *v* 52.0% | 1.000 | 67.9% *v* 45.8% | **0.007** | 73.7% *v* 62.8% | 0.154 |
| IPSS-R, LR *v* HR (37 *v* 69) | 62.2% *v* 49.3% | 0.226 | 77.4% *v* 53.0% | **0.039** | 81.4% *v* 65.5% | **0.044** |
| HMA, AZA *v* DAC (66 *v* 41) | 60.6% *v* 41.5% | 0.073 | 61.3% *v* 63.3% | 0.298 | 69.1% *v* 74.8% | 0.813 |
| Gene mutations (no *v* yes) |  |  |  |  |  |  |
| Methylation |  |  |  |  |  |  |
| *TET2* (90 *v* 17) | 53.3% *v* 52.9% | 1.000 | 63.3% *v* 55.8% | 0.941 | 71.6% *v* 67.7% | 0.707 |
| *DNMT1* (102 *v* 5) | 54.9% *v* 20.0% | 0.183 | 64.5% *v* 20.0% | **0.012** | 72.2% *v* 50.0% | 0.252 |
| *DNMT3A* (98 *v* 9) | 54.1% *v* 44.4% | 0.731 | 67.0% *v* 13.9% | **0.001** | 76.4% *v* 14.8% | **2.1x10^-7^** |
| *IDH1* (104 *v* 3) | 52.9% *v* 66.7% | 1.000 | 62.2% *v* 66.7% | 0.723 | 71.3% *v* 66.7% | 0.843 |
| *IDH2* (106 *v* 1) | 52.8% *v* 100% | 1.000 | 62.1% *v* 100% | 0.520 | 71.0% *v* 100% | 0.572 |
| *IDH1/IDH2* (103 *v* 4*)* | 52.4% *v* 75.0% | 0.621 | 61.9% *v* 75.0% | 0.533 | 71.0% *v* 75.0% | 0.894 |
| Chromatin modulation |  |  |  |  |  |  |
| *ASXL1* (87 *v* 20) | 54.0% *v* 50.0% | 0.807 | 61.7% *v* 65.0% | 0.586 | 71.3% *v* 72.1% | 0.661 |
| *EZH2* (97 *v* 10) | 52.6% *v* 60.0% | 0.748 | 60.0% *v* 90.0% | 0.241 | 70.8% *v* 75.0% | 0.670 |
| *STAG2* (104 *v* 3) | 53.8% *v* 33.3% | 0.598 | 62.9% *v* 50.0% (1-y) | 0.120 | 72.7% *v* 0% (1-y) | **9.1x10^-8^** |
| *ATRX* (100 *v* 7) | 53.0% *v* 57.1% | 1.000 | 61.0% *v* 85.7% | 0.278 | 69.4% *v* 100% | 0.150 |
| Splicing |  |  |  |  |  |  |
| *U2AF1* (86 *v* 21) | 58.1% *v* 33.3% | 0.052 | 61.6% *v* 65.0% | 0.903 | 68.5% *v* 85.0% | 0.300 |
| *SF3B1* (96 *v* 11) | 52.1% *v* 63.6% | 0.537 | 63.6% *v* 53.0% | 0.214 | 70.9% *v* 74.1% | 0.972 |
| *ZRSR2* (102 *v* 5) | 52.9% *v* 60.0% | 1.000 | 61.5% *v* 80.0% | 0.787 | 69.9% *v* 100% | 0.212 |
| *SRSF2* (106 *v* 1) | 52.8% *v* 100% | 1.000 | 62.0% *v* 100% | 0.511 | 70.9% *v* 100% | 0.559 |
| *PRPF8* (105 *v* 2) | 54.3% *v* 0% | 0.216 | 61.8% *v* 100% | 0.339 | 70.8% *v* 100% | 0.436 |
| Transcription |  |  |  |  |  |  |
| *RUNX1* (95 *v* 12) | 53.7% *v* 50.0% | 1.000 | 64.5% *v* 47.6% | 0.525 | 71.3% *v* 71.4% | 0.922 |
| *TP53* (94 *v* 13) | 50.0% *v* 76.9% | 0.082 | 67.0% *v* 30.8% | **0.003** | 73.6% *v* 53.3% | 0.074 |
| *ETV6* (100 *v* 7) | 55.0% *v* 28.6% | 0.248 | 62.3% *v* 62.5% | 0.676 | 71.7% *v* 64.3% | 0.842 |
| *NPM1* (106 *v* 1) | 53.8% *v* 0% | 0.467 | 63.0% *v* 0% | **0.029** | 72.0% *v* 0% (1-y) | **0.042** |
| RAS pathway |  |  |  |  |  |  |
| *NRAS* (98 *v* 9) | 53.1% *v* 55.6% | 1.000 | 67.1% *v* 0% | **3.4x10^-4^** | 75.8% *v* 0% | **6.2x10^-5^** |
| *CBL* (101 *v* 6) | 54.5% *v* 33.3% | 0.415 | 63.1% *v* 50.0% | 0.669 | 71.6% *v* 66.7% | 0.650 |
| *NF1* (99 *v* 8) | 54.5% *v* 37.5% | 0.469 | 62.3% *v* 62.5% | 0.237 | 70.2% *v* 85.7% | 0.460 |
| *KRAS* (103 *v* 4) | 53.4% *v* 50.0% | 1.000 | 63.5% *v* 0% (1-y) | **0.001** | 71.7% 75.0% (1-y) | 0.237 |
| *NRAS/KRAS* (96 *v* 11) | 54.2% *v* 45.5% | 0.752 | 67.8% *v* 0% | **1.9x10^-5^** | 76.6% *v* 0% | **1.5x10^-6^** |
| Other |  |  |  |  |  |  |
| *LAMB4* (102 v 5) | 53.9% *v* 40.0% | 0.663 | 61.4% *v* 80.0% | 0.931 | 70.9% *v* 80.0% | 0.933 |
| *SETBP1* (103*v* 4) | 55.3% *v* 0% | **0.045** | 61.9% *v* 75.0% | 0.395 | 72.0% *v* 50.0% | 0.229 |
| *JAK2* (100 *v* 7) | 52.0% *v* 71.4% | 0.445 | 60.6% *v* 85.7% | 0.563 | 70.0% *v* 85.7% | 0.818 |
| *FLT3* (105 *v* 2) | 54.3% *v* 0*%* | 0.216 | 62.8% *v* 50.0% (1-y) | 0.181 | 71.8% *v* 50.0% (1-y) | 0.163 |

HMT, hypomethylating therapy; AML, acute myelogenous leukemia; ANC, absolute neutrophil count; Hb, hemoglobin; PLT, platelet; IPSS-R, revised International Prognostic Scoring System; VG, Very Good; G, Good; INT, Intermediate; P, Poor; VP, Very Poor; LR, lower-risk; HR, higher-risk; HMA, hypomethylating agent; AZA, azacitidine; DAC, decitabine

Bold, *P*<0.05
